# Human umbilical cord mesenchymal stem cell–derived exosomes are associated with changes in renal injury markers, gut microbiota composition, and inflammatory signaling in IgA nephropathy

**DOI:** 10.3389/fimmu.2026.1854005

**Published:** 2026-06-01

**Authors:** Yuanyuan He, Xinyi Wang, Qian Hu, Lu Huang, Shibin Zhang, Shengzhi Zhang, Zhengxia Zhong

**Affiliations:** 1 Department of Nephrology, Affiliated Hospital of Zunyi Medical University, Zunyi, Guizhou, China; 2 Laboratory Animal Centers, Zunyi Medical University, Zunyi, China; 3 Department of Nephrology, Guizhou Moutai Hospital, Zunyi, Guizhou, China

**Keywords:** gut microbiota, gut–kidney axis, IgA nephropathy, mesenchymal stem cell–derived exosomes, NLRP3 inflammasome

## Abstract

**Background:**

IgA nephropathy (IgAN) is the most common primary glomerulonephritis worldwide and a leading cause of end-stage kidney disease, yet disease-specific therapeutic options remain limited. Emerging evidence implicates gut microbiota dysbiosis and innate immune activation, particularly NLRP3 (NOD-, LRR- and pyrin domain-containing protein 3) inflammasome-related signaling, in IgAN pathogenesis. However, whether human umbilical cord mesenchymal stem cell–derived exosomes (hUCMSC-Exos) are associated with changes in renal injury and gut–immune-related parameters in IgAN remains unclear.

**Methods:**

hUCMSC-Exos were isolated and administered to an IgAN-like mouse model. Renal function, histopathological changes, and systemic inflammatory markers were assessed. Gut microbiota composition was analyzed using 16S rRNA sequencing, and exploratory microbial co-occurrence networks were constructed. *In vitro*, podocytes stimulated with galactose-deficient IgA1 (Gd-IgA1) were used to evaluate inflammasome-related markers following exosome exposure. Transcriptomic data from human IgAN glomeruli (GSE93798) were analyzed to explore inflammatory and immune-related gene signatures.

**Results:**

hUCMSC-Exos were associated with changes in renal injury markers in IgAN-like mice, along with alterations in gut microbial composition. Microbiome analysis showed a shift toward a microbial profile closer to controls, with enrichment of bacterial taxa previously reported in association with gut metabolic homeostasis in other cohorts, including Anaerostipes, Dorea, and Ruminococcus. These taxa showed correlations with renal dysfunction indicators and inflammatory markers and were identified as hub taxa in an exploratory co-occurrence network. Transcriptomic analysis of human IgAN glomeruli revealed altered expression of NLRP3 inflammasome-related genes and aryl hydrocarbon receptor (AhR)-related signaling components, suggesting context-dependent inflammatory activity requiring further validation. *In vitro*, hUCMSC-Exos were associated with reduced levels of NLRP3, IL-1β, and IL-18 in Gd-IgA1–stimulated podocytes.

**Conclusions:**

hUCMSC-Exos were associated with changes in renal injury markers in an IgAN-like model, accompanied by alterations in gut microbiota composition and inflammasome-related inflammatory markers. These findings are consistent with a potential association between gut microbiota, innate immune-related signaling, and renal injury in IgAN. hUCMSC-Exos may represent a cell-free candidate for further investigation in IgAN. However, these observations are descriptive and associative in nature, and causal mechanisms cannot be inferred from the present study.

## Introduction

IgA nephropathy (IgAN) is the most prevalent primary glomerulonephritis worldwide and a major cause of end-stage renal disease (1, 2). Despite advances in supportive care, disease-specific therapeutic strategies remain limited, and progressive renal dysfunction is common. A central pathogenic feature of IgAN is the mesangial deposition of galactose-deficient IgA1 (Gd-IgA1), which triggers mesangial cell activation, inflammatory responses, and subsequent renal fibrosis (3, 4).

The gut−renal connection in IgAN has been extensively reviewed (5). Increasing evidence highlights a critical role of the gut–kidney axis in the pathogenesis of IgAN (6, 7). Gut microbiota dysbiosis has been associated with mucosal immune imbalance, systemic inflammation, and altered microbial metabolic activity, particularly involving short-chain fatty acid (SCFA)–related pathways (8, 9). Our previous clinical studies demonstrated that patients with IgAN exhibit reduced abundance of butyrate-producing bacteria, accompanied by systemic inflammatory activation, including elevated circulating levels of NLRP3 (NOD-, LRR- and pyrin domain-containing protein 3) inflammasome components and pro-inflammatory cytokines such as IL-18 and IL-6 (10, 11). Furthermore, recent studies have linked specific microbial taxa to disease severity and reported decreased fecal SCFA levels in IgAN patients (12–14). Collectively, these findings suggest an association between gut dysbiosis and innate immune activation in IgAN; however, the underlying mechanisms remain incompletely understood. A recent bidirectional Mendelian randomization study has provided genetic evidence supporting causal links between specific gut microbes and IgAN (15).

The aryl hydrocarbon receptor (AhR) is a ligand-activated transcription factor that responds to a wide range of endogenous, dietary, and microbiota-derived metabolites, particularly those derived from tryptophan metabolism (16–19). In addition to its role in xenobiotic sensing, AhR has emerged as an important regulator of mucosal immune homeostasis and host–microbiota interactions (17). Notably, AhR signaling has been suggested to interact with inflammatory pathways, including NLRP3 inflammasome-related responses (20, 21). However, its functional relevance within the gut–kidney axis in IgAN remains unclear, and whether microbiota-associated AhR signaling contributes to inflammasome regulation in this context requires further investigation.

Mesenchymal stem cell–derived exosomes (MSC-Exos) have emerged as promising cell-free therapeutic agents due to their immunomodulatory and tissue-reparative properties (22–24). Human umbilical cord MSC-derived exosomes (hUCMSC-Exos) have demonstrated renoprotective effects in experimental kidney diseases (25–28). For instance, hUCMSC-Exos ameliorate diabetic kidney disease by inhibiting podocyte epithelial-mesenchymal transition (EMT) (27) and suppress renal fibrosis via macrophage modulation (28). Nevertheless, whether hUCMSC-Exos are associated with changes in gut microbiota composition and inflammasome-related signaling in podocytes in the context of IgAN remains unknown. In this study, we investigated whether hUCMSC-Exos are associated with coordinated changes in gut microbiota and NLRP3 inflammasome activity. To test this hypothesis, we integrated an IgAN mouse model, microbiome profiling, *in vitro* podocyte assays, and analysis of human kidney transcriptomic data (GSE93798).

## Materials and methods

### Animals and ethics

SPF-grade male and female C57BL/6 mice (6–8 weeks old, 20 ± 5 g) were obtained from the Animal Experiment Center of Zunyi Medical University (license No. SCXK (Qian) 2021-0002). All animal procedures were approved by the Ethics Committee of Zunyi Medical University (approval No. zyfy-an-2024-0319) and conducted in accordance with institutional guidelines for the care and use of laboratory animals.

### Cells and reagents

Mouse podocytes were kindly provided by Professor Wei Qin (Sichuan University). Human umbilical cord mesenchymal stem cells (hUCMSCs) were obtained from Professor Limei Yu (Zunyi Medical University). Gd-IgA1 was isolated from serum of IgAN patients using Jacalin-agarose affinity chromatography and verified by ELISA.

Primary antibodies against CD81, ALIX, Calnexin (Immunoway), NLRP3 (Novus Biologicals), and GAPDH were used. ELISA kits for IL-1β, IL-18, NLRP3, serum creatinine (Scr), and blood urea nitrogen (BUN) were purchased from Shanghai Enzyme-linked Biotechnology.

### Cell culture and treatment

Mouse podocytes were divided into three groups: control, Gd-IgA1, and Gd-IgA1+hUCMSC-Exos. The Gd-IgA1 group was stimulated with Gd-IgA1 (1 mg/mL) for 12 h, and the Gd-IgA1+hUCMSC-Exos group was co-treated with Gd-IgA1 (1 mg/mL) and hUCMSC-Exos (1 × 10^9^ particles/mL) for 12 h. Control cells received vehicle only. Cells were cultured in RPMI-1640 medium supplemented with standard additives in 24-well plates (500 μL per well). All *in vitro* experiments were performed with three independent biological replicates, each with three technical replicates.

### Isolation and characterization of hUCMSC-Exos

hUCMSCs were cultured in exosome-depleted medium under serum-free conditions and exhibited a typical fibroblast-like morphology. Cells expressed mesenchymal markers CD73, CD90, and CD105, and were negative for CD45.

When passage-5 hUCMSCs reached approximately 70% confluence, the medium was replaced with exosome-depleted medium, and cells were cultured for an additional 48h. hUCMSC-Exos were isolated by differential ultracentrifugation according to MISEV2023 guidelines (29).

Exosome size distribution and concentration were analyzed by nanoparticle tracking analysis (NTA), and morphology was assessed by transmission electron microscopy (TEM). Expression of exosomal markers CD9, CD81, and ALIX, as well as absence of Calnexin, was confirmed by Western blotting. Exosomes were aliquoted and stored at −80 °C and thawed only once before use. All exosome characterization experiments were performed in at least three independent biological replicates to ensure reproducibility.

For *in vivo* experiments, mice received 1 × 10^9^ particles per injection; for *in vitro* experiments, cells were treated with 1 × 10^9^ particles/mL. Detailed characterization parameters such as endotoxin levels and particle-to-protein ratio were not assessed and represent a limitation of this study.

### IgAN mouse model and exosome treatment

IgAN was induced using a combined protocol consisting of: (i) bovine serum albumin (BSA, 400 mg/kg by gavage every other day for 6 weeks); (ii) carbon tetrachloride/ricinus oil mixture (2:3 v/v, CCl_4_ concentration 10% in olive oil) injected subcutaneously in the interscapular region at 0.1 mL per mouse weekly for 8 weeks; and (iii) lipopolysaccharide (LPS, 0.02 mg, approximately 0.8 mg/kg) administered via tail vein injection at weeks 6 and 8. All procedures were performed in a fume hood with appropriate personal protective equipment. Control mice received saline. This model reproduces selected features of human IgAN, including mesangial IgA deposition and proteinuria.

The model induction phase was completed before initiation of exosome treatment; however, formal baseline validation before randomization was not performed. Mice were randomly assigned to IgAN and IgAN+hUCMSC-Exos groups (n = 5 per group). Each group contained 3 males and 2 females. Blinding was not implemented during experiments or outcome assessment. Exosomes (1 × 10^9^ particles in 100 μL PBS) were administered via tail vein injection twice weekly for 4 weeks. Renal function was assessed by serum creatinine (Scr), blood urea nitrogen (BUN), urinary protein-to-creatinine ratio (UPCR), and serum inflammatory markers. Kidney tissues were analyzed by HE and PAS staining, and by immunofluorescence for IgA deposition.

### Gut microbiota analysis

Fecal samples were collected at week 14 (after 4 weeks of exosome treatment) between 9:00 and 10:00 AM, immediately after metabolic cage urine collection. Fecal DNA was extracted and the V3–V4 region of bacterial 16S rRNA genes was sequenced using the Illumina NovaSeq 6000 platform. Raw reads were processed using QIIME2 (version 2022.2) with the DADA2 plugin for quality filtering and denoising. To account for uneven sequencing depth, the feature table was rarefied to an even depth of 27,018 reads per sample, which retained the majority of samples and provided sufficient coverage as assessed by rarefaction curves (**Supplementary Figure 2**). Taxonomic assignment was performed against the SILVA 138 database.

Alpha diversity (Shannon and Chao1 indices) and beta diversity (Bray–Curtis distance, PCoA, PERMANOVA with 9,999 permutations, and pairwise PERMANOVA with Bonferroni correction) were analyzed using the Shanghai Majorbio bioinformatics platform (https://www.majorbio.com). Differential abundance of genera was assessed by Kruskal-Wallis tests followed by Benjamini-Hochberg false discovery rate (FDR) correction; q < 0.05 was considered significant.

An exploratory microbial co-occurrence network was constructed based on Spearman’s rank correlations (|r| > 0.6, P < 0.05). Only statistically significant correlations were included to generate the adjacency matrix, and the network was visualized using Gephi (v0.9.2). These analyses reflect potential ecological associations and do not imply causality. Given the compositional nature of 16S rRNA gene sequencing data and the limited sample size, all microbiome analyses are interpreted as associative and exploratory. Advanced compositional data analysis methods (e.g., ANCOM-BC or ALDEx2) were not applied; therefore, results should be interpreted with caution. All 16S sequencing was performed on one sample per animal (n=5 per group), and no technical replicates were performed.

### Podocyte experiments and *in vitro* assays

Podocytes were divided into control, Gd-IgA1, and Gd-IgA1+hUCMSC-Exos groups. Cell viability was assessed using CCK-8 assay. Supernatants were collected for measurement of IL-1β, IL-18, and NLRP3 by ELISA.

### Western blot and RT-qPCR

Total protein was extracted using RIPA buffer and quantified by BCA assay. Proteins were separated by SDS-PAGE, transferred to PVDF membranes, and incubated with primary antibodies overnight at 4 °C. Detection was performed using enhanced chemiluminescence (ECL), and band intensity was quantified using ImageJ.

Total RNA was extracted using TRIzol reagent, reverse-transcribed, and analyzed by RT-qPCR. Relative gene expression was calculated using the 2^-^ΔΔCt method with GAPDH as internal control. Western blotting was repeated three times with independent lysates.

### Human kidney transcriptome analysis (GSE93798)

The GSE93798 dataset (22 controls and 20 IgAN patients) was downloaded from GEO. Data were normalized, and probe sets were mapped to gene symbols using GPL22945 annotation.

Differential expression analysis was performed using the Wilcoxon rank-sum test with Benjamini–Hochberg correction. An NLRP3 inflammasome signature (NLRP3, PYCARD, CASP1, IL1B, IL18) was defined, and the signature score was calculated as the mean expression of these genes.

### Statistical analysis

Data are presented as mean ± SD or median (IQR), as appropriate. Normality of continuous variables was assessed using the Shapiro–Wilk test, and homogeneity of variances was checked using Levene’s test. For normally distributed data with equal variances, two-group comparisons were performed using Student’s t-test; otherwise, the Welch t-test was used. For non-normally distributed data, the Wilcoxon rank-sum test was applied. Multiple-group comparisons were analyzed using one-way ANOVA with Tukey’s *post hoc* test (parametric, equal variance) or the Kruskal–Wallis test with Dunn’s *post hoc* test and Bonferroni correction (non-parametric). For microbiome differential abundance, p-values from Kruskal–Wallis tests were adjusted using the Benjamini–Hochberg false discovery rate (FDR) method; q < 0.05 was considered significant. Spearman correlation was used for association analysis. All statistical tests were two-tailed, and a P-value < 0.05 was considered statistically significant unless otherwise specified. Most statistical analyses were performed using R software (version 4.3.2) and GraphPad Prism (version 9.0). Microbiome analyses (alpha diversity, beta diversity, PERMANOVA, and pairwise PERMANOVA) were performed using the Shanghai Majorbio bioinformatics platform (https://www.majorbio.com).

## Results

### hUCMSC-Exos are associated with gut microbiota changes and enrichment of taxa previously linked to SCFA-producing communities

**Figure 1:**
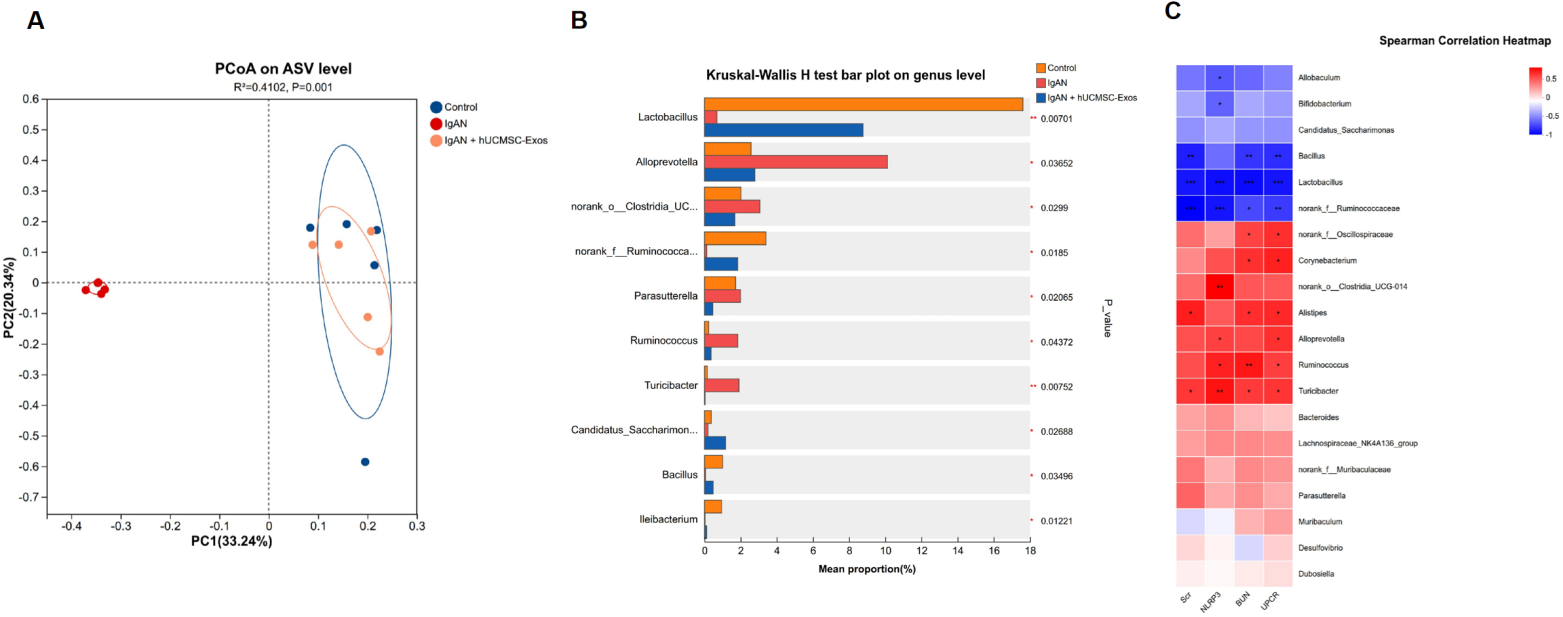
hUCMSC-Exos modulate gut microbiota composition in IgAN-like mice. **(A)** Principal coordinate analysis (PCoA) based on Bray-Curtis distance showing microbial community structure among Control (n=5), IgAN (n=5), and IgAN+Exos (n=5) groups. PERMANOVA: R² = 0.4102, P = 0.001. **(B)** Genera previously associated with differentially enriched taxa in other studies are highlighted. **(C)** Spearman correlation analysis between representative genera and renal function/inflammatory parameters (Scr, UPCR, BUN, and serum NLRP3). Correlations are exploratory; uncorrected P values are shown. Data are presented as mean ± SD. *P < 0.05, **P < 0.01, ***P < 0.001 (Kruskal-Wallis test with Dunn’s *post-hoc* for B; Spearman correlation for C). Details are provided in Methods.

Principal coordinate analysis (PCoA) based on Bray–Curtis distance demonstrated a clear separation among control, IgAN, and hUCMSC-Exos–treated groups (PERMANOVA, R² = 0.4102, P = 0.001), with the treatment group shifting toward the control cluster (**Figure 1A**). Alpha diversity, assessed by Shannon and Chao1 indices, did not differ significantly among groups (**Supplementary Figures 3A, B**; Shannon: P = 0.2982; Chao1: P = 0.4025).

Pairwise PERMANOVA with Bonferroni correction showed a significant difference between the Control and IgAN groups (adjusted P = 0.03), whereas the difference between IgAN and IgAN+hUCMSC-Exos did not reach statistical significance (adjusted P = 0.066), although a compositional shift toward a control-like microbial community structure was observed in PCoA space (**Figure 1A**; **Supplementary Table 2**).

Differential abundance analysis identified nominal changes in several genera prior to FDR correction (**Supplementary Table 1**), with a trend toward enrichment of bacterial taxa previously associated with gut metabolic homeostasis, including Lactobacillus and Bacillus, and a reduction in opportunistic pathogens such as Alloprevotella and Turicibacter (**Figure 1B**).

Spearman correlation analysis showed that genera previously associated with SCFA-related microbial communities were negatively correlated with renal dysfunction parameters (Scr, UPCR, BUN) and serum NLRP3 levels, whereas putative pathogenic taxa exhibited opposite trends (**Figure 1C**).

**Figure 2:**
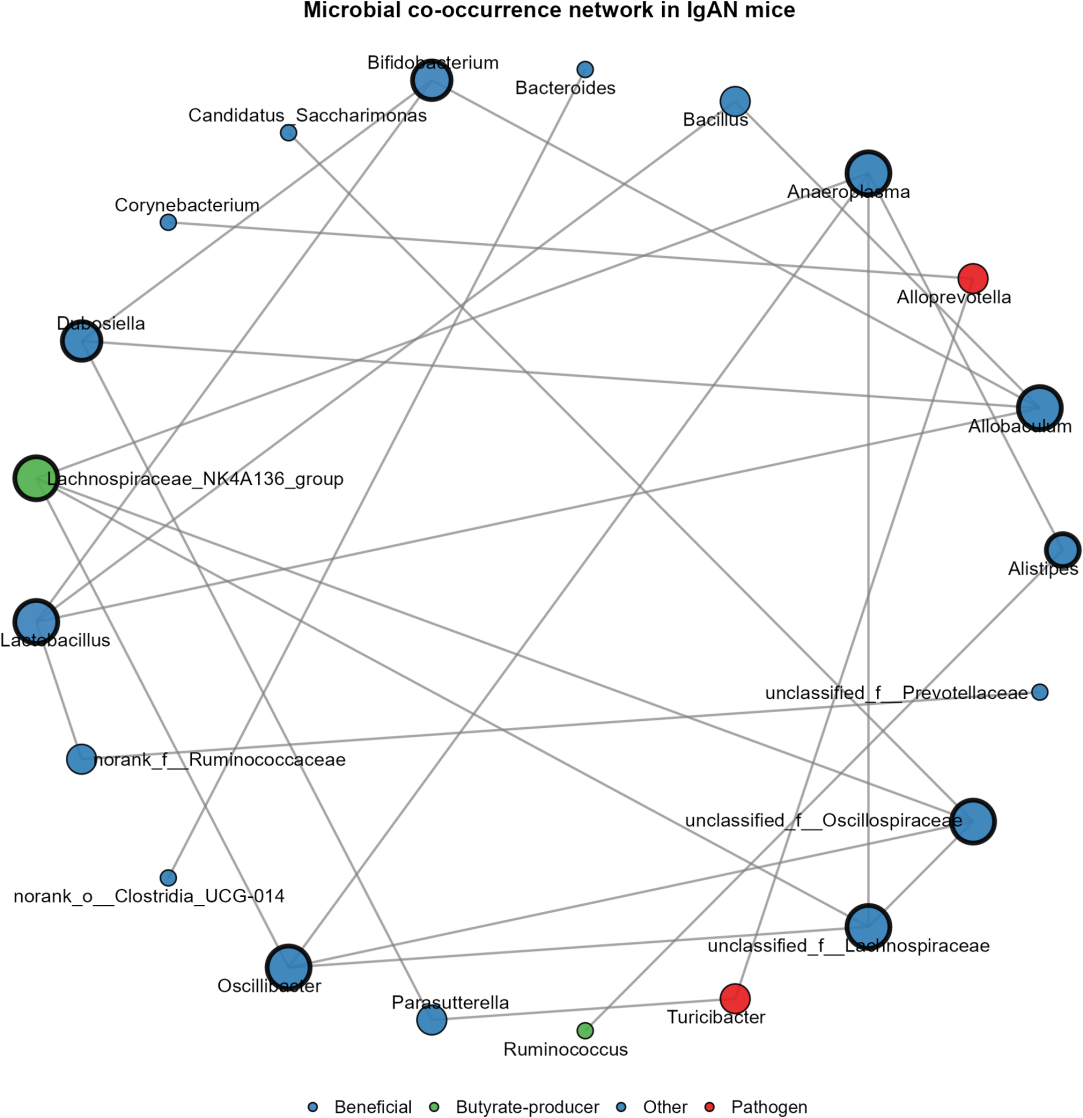
Exploratory microbial co−occurrence network. Network constructed based on Spearman correlation (|r| > 0.6, P < 0.05) using genus-level relative abundances. Because correlation-based methods do not account for the compositional nature of 16S sequencing data, this network should be interpreted as exploratory and hypothesis−generating. Nodes represent bacterial genera; edges represent significant correlations. Hub taxa (degree > 10) are highlighted. No causal inferences can be drawn from this analysis.

In the exploratory co-occurrence network, taxa previously associated with gut metabolic activity and SCFA-related microbial profiles appeared as highly connected nodes (**Figure 2**). However, given the limitations of correlation-based network inference and 16S rRNA gene sequencing, these observations reflect potential ecological associations only and should not be interpreted as functional or mechanistic relationships. These analyses are exploratory and do not imply causality.

### Validation of the AhR–NLRP3 axis in human IgAN transcriptomic data

**Figure 3:**
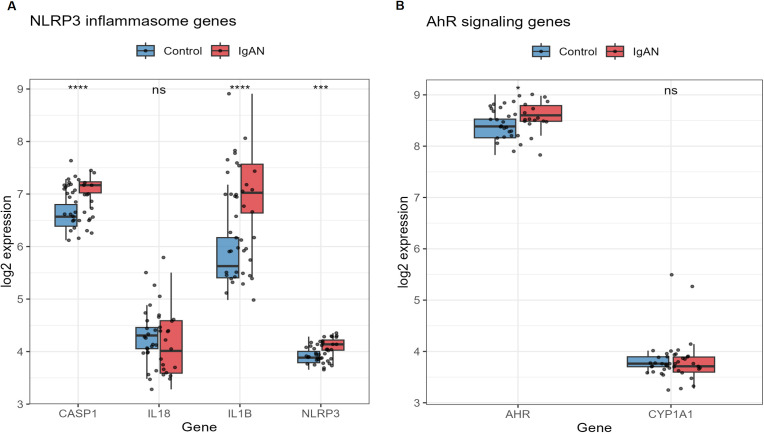
NLRP3 inflammasome-related gene expression in human IgAN glomeruli (GSE93798). **(A)** Differential expression of NLRP3 inflammasome-related genes (NLRP3, CASP1, IL1B, IL18) between IgAN patients (n=20) and healthy controls (n=22). **(B)** Expression of AhR and CYP1A1. Data are presented as mean ± SD. *P < 0.05, **P < 0.01, ***P < 0.001 (Wilcoxon rank-sum test with Benjamini-Hochberg correction).

Analysis of the GSE93798 dataset revealed significant upregulation of NLRP3 inflammasome-related genes, including NLRP3, CASP1, and IL-1β, in IgAN patients compared with controls, whereas IL-18 showed no significant difference (**Figure 3A**). AhR expression was increased, while its canonical downstream target CYP1A1 remained unchanged (**Figure 3B**).

### hUCMSC-Exos are associated with reduced renal dysfunction and histopathological injury in IgAN-like mice

**Figure 4:**
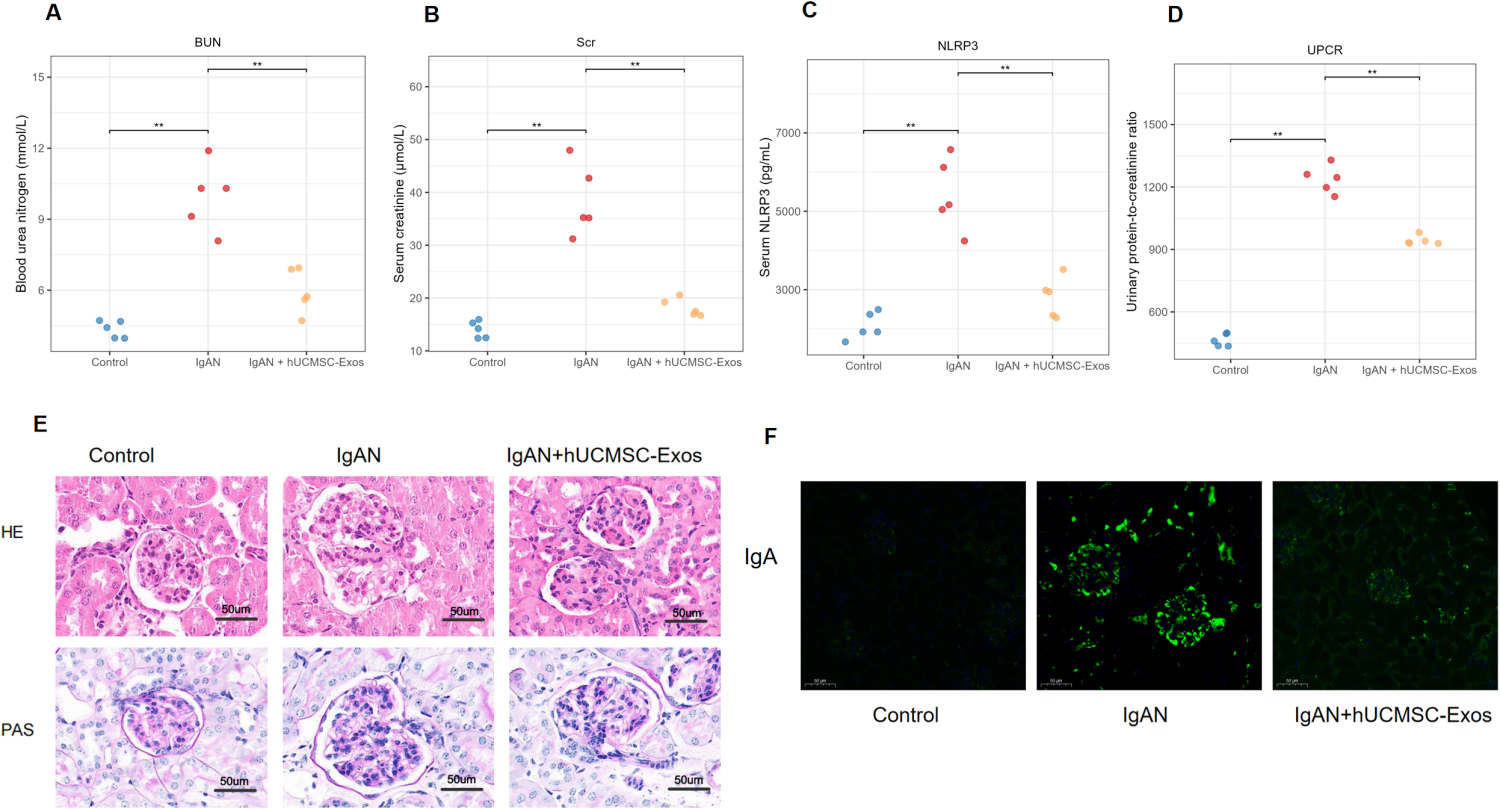
hUCMSC-Exos are associated with reduced renal dysfunction and histopathological injury in IgAN-like mice. **(A)** Blood urea nitrogen (BUN). **(B)** Serum creatinine (Scr). **(C)** Serum NLRP3 levels. **(D)** Urinary protein-to-creatinine ratio (UPCR). **(E)** Representative histological images are shown; quantitative histopathological scoring could not be performed due to limited remaining tissue availability. **(F)** Immunofluorescence analysis of glomerular IgA deposition. Data are presented as mean ± SD. *P < 0.05, **P < 0.01, ***P < 0.001 (one-way ANOVA with Tukey’s *post hoc* test for normally distributed data; otherwise Kruskal–Wallis test with Dunn’s correction). Each dot represents one animal.

IgAN-like mice exhibited significantly increased blood urea nitrogen (BUN) (**Figure 4A**), serum creatinine (Scr) (**Figure 4B**), serum NLRP3 levels (**Figure 4C**), and urinary protein-to-creatinine ratio (UPCR) (**Figure 4D**) compared with controls (all P < 0.01). Treatment with hUCMSC-Exos significantly reduced these parameters (all P < 0.05), with values approaching those of the control group. Representative histological analyses suggested that hUCMSC-Exos treatment may attenuate mesangial expansion and inflammatory features; quantitative scoring could not be performed due to limited tissue availability (**Figure 4E**). Immunofluorescence staining further demonstrated reduced mesangial IgA deposition in the treatment group (**Figure 4F**).

### hUCMSC-Exos are associated with reduced inflammasome-related markers in podocytes

**Figure 5:**
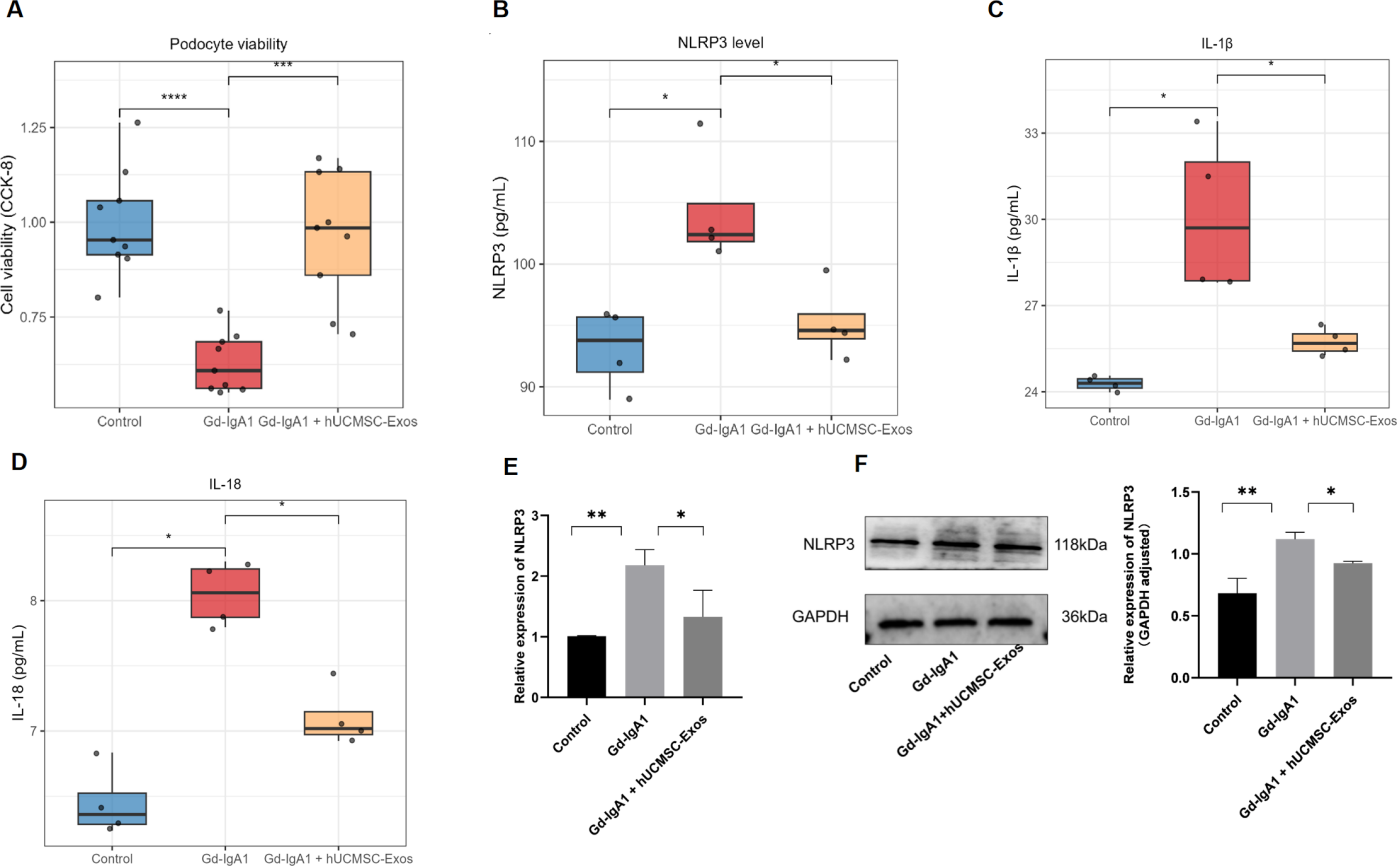
hUCMSC-Exos are associated with reduced podocyte injury and NLRP3 inflammasome-related responses *in vitro*. **(A)** Podocyte viability assessed by CCK-8 assay. **(B–D)** Levels of IL-1β, IL-18, and NLRP3 in culture supernatants measured by ELISA. **(E)** NLRP3 mRNA expression analyzed by RT-qPCR. **(F)** Representative Western blot analysis of NLRP3 protein expression (n=3 independent experiments). All *in vitro* experiments were performed with three independent biological replicates, each with three technical replicates. Data are presented as mean ± SD. *P < 0.05, **P < 0.01, ***P < 0.001 (one-way ANOVA with Tukey’s *post-hoc* test).

*In vitro*, Gd-IgA1 stimulation significantly reduced podocyte viability, whereas co-treatment with hUCMSC-Exos partially restored cell viability (**Figure 5A**). Gd-IgA1 markedly increased the levels of NLRP3, IL-1β, and IL-18 in culture supernatants (**Figures 5B–D**), as well as NLRP3 mRNA expression (**Figure 5E**), which were significantly attenuated by exosome treatment. Western blot analysis further confirmed the downregulation of NLRP3 protein expression (**Figure 5F**).

## Discussion

In this study, we provide multi-level evidence suggesting that hUCMSC-Exos are associated with renoprotective effects in IgAN. By integrating *in vivo*, *in vitro*, and human transcriptomic analyses, our findings indicate that hUCMSC-Exos are associated with improved renal function and attenuated histopathological injury, along with modulation of gut microbiota composition and changes in inflammasome-related inflammatory responses in podocytes. However, the present study does not directly compare exosomes with other therapeutic modalities, and therefore their relative therapeutic efficacy remains to be established. Collectively, these findings suggest involvement of gut–kidney–immune axis–related processes in the pathogenesis and progression of IgAN (30, 31). Importantly, the present data do not establish whether microbiota changes or inflammasome modulation are required for the observed renal protection.

### Renoprotective effects of hUCMSC-Exos

Our *in vivo* data suggest that hUCMSC-Exos administration was associated with significant improvement in renal functional parameters, including Scr, BUN, and UPCR, suggested attenuation of mesangial expansion and IgA deposition; quantitative analysis was not performed due to limited tissue availability (**Figures 4A–F**). These findings are consistent with previous studies reporting the therapeutic potential of hUCMSC-Exos in kidney diseases, particularly through anti-inflammatory and tissue-protective potential mechanisms (22–24, 27, 28). In addition, exosome-associated biomarkers have been investigated in IgAN, further supporting their potential clinical relevance (25, 26). Although the underlying potential mechanisms remain incompletely defined, our results are consistent with exosome-based strategies as a potential cell-free therapeutic approach for IgAN.

### Potential involvement of NLRP3 inflammasome-related changes

At the cellular level, our *in vitro* experiments indicate that hUCMSC-Exos may attenuate Gd-IgA1–induced podocyte injury, at least in part, in association with changes in inflammasome-related signaling markers (**Figures 5A–F**). This is supported by reduced expression of NLRP3, IL-1β, and IL-18, together with partial restoration of podocyte viability following treatment. These results are consistent with previous studies implicating NLRP3 inflammasome-related responses in podocyte injury and chronic kidney inflammation (32, 33). In addition, gut microbiota modulation has been reported to influence NLRP3 inflammasome activity in IgAN, suggesting a broader role of this pathway in disease pathophysiology (34). However, in the absence of genetic or pharmacological inhibition experiments, these findings do not establish causality. In addition, key markers of inflammasome-related responses such as cleaved caspase-1, ASC oligomerization, or GSDMD cleavage were not assessed, and therefore inflammasome-related responses cannot be definitively established. Therefore, NLRP3 inflammasome involvement should be interpreted as a putative mechanistic association rather than a confirmed causal pathway.

### Gut microbiota remodeling and ecological implications

Our microbiome analysis revealed that hUCMSC-Exos were associated with changes in gut microbial composition toward a control-like microbial composition (**Figures 1A–C**). This shift was characterized by enrichment of bacterial genera such as Anaerostipes, Dorea, and Ruminococcus, which have been previously linked to short-chain fatty acid (SCFA)-producing microbial communities in other studies (8, 9, 14). Correlation analyses further demonstrated that these taxa were negatively associated with renal dysfunction markers and inflammatory indicators (**Figure 1C**), while network analysis suggested their contribution to microbial community stability (**Figure 2**). Although some of these taxa have been associated with SCFA-associated microbial communities in previous studies, these functional interpretations remain speculative due to the limitations of 16S rRNA sequencing. These findings are consistent with previous reports linking gut dysbiosis to disease severity in IgAN (6, 8, 10). However, as 16S rRNA sequencing does not provide functional or metabolic resolution, the biological activity of these microbial changes remains inferential and requires further validation using metabolomic or functional approaches. A comparative study reported distinct gut microbial signatures between IgAN and membranous nephropathy (MN) (35). While both diseases exhibit gut dysbiosis, certain taxa such as Lactobacillus and Bifidobacterium were differentially altered. Our hUCMSC-Exos treatment enriched Lactobacillus and Ruminococcus, but the specificity of our findings to IgAN remains speculative. Head−to−head comparative studies are needed.

The potential of MSC-based therapies to modulate gut microbiota and immune responses has also been explored in other autoimmune kidney diseases. For instance, a recent study found that early MSC transplantation extended therapeutic benefits in lupus-prone mice, an effect linked to the modulation of gut microbiota composition (36). Furthermore, engineering MSC-derived extracellular vesicles (EVs) within an inflammatory microenvironment has been shown to enhance their immunomodulatory efficacy in treating SLE (37). These observations, while in a different disease context, support the broader potential of MSC-EV-based strategies in modulating the gut–kidney–immune axis in autoimmune renal diseases, providing relevant context for our findings in IgAN.

### Correlation with clinical stages

In a Chinese IgAN cohort, gut microbiota modifications correlated with clinical severity, including reduced Bifidobacterium and increased Escherichia−Shigella associated with higher proteinuria and Oxford MEST scores (10). While our mouse model does not recapitulate human clinical stages, we observed that taxa enriched by hUCMSC-Exos (e.g., Ruminococcus, Lactobacillus) were negatively correlated with proteinuria and serum NLRP3 levels. Direct validation in staged patient cohorts is required.

### Putative involvement of microbial metabolites associated with SCFA-producing taxa

The enrichment of bacterial genera with SCFA-related metabolic profiles raises the possibility that microbial metabolites may contribute to the immunomodulatory effects observed in this study. Butyrate, in particular, has been proposed in previous studies to regulate inflammatory responses and epigenetic processes, including histone deacetylase inhibition, thereby influencing immune homeostasis and tissue integrity (38–42). In kidney diseases, SCFAs have been implicated in anti-inflammatory and anti-fibrotic effects, suggesting a potential role in maintaining renal homeostasis (39, 40, 43). Moreover, reduced fecal SCFA levels have been reported in patients with IgAN, supporting a possible association with disease pathophysiology (14). However, as SCFA concentrations were not directly measured, their functional involvement remains hypothetical. Future metabolomic and functional studies are required to clarify their roles in the gut–kidney axis.

### Context-dependent AhR signaling in IgAN

Analysis of human IgAN transcriptomic data (GSE93798) revealed altered expression of genes associated with both NLRP3 inflammasome signaling and AhR-related pathways (**Figures 3A, B**). Although AhR expression was increased, its canonical downstream target CYP1A1 did not show corresponding changes. Because GSE93798 contains bulk glomerular transcriptomic data, the observed expression changes cannot distinguish intrinsic renal-cell signaling from immune-cell infiltration, suggesting a potential context-dependent or non-canonical pattern of AhR-related signaling in IgAN. AhR has been reported to participate in immune regulation and inflammatory signaling networks, including interactions with NF-κB and inflammasome-related pathways (15, 18, 20). In addition, microbiota-derived tryptophan metabolites are known ligands of AhR (19), which provides a theoretical basis for a possible association between microbial metabolism and host immune signaling. However, in the absence of functional experiments or metabolite measurements, these transcriptomic observations cannot establish causality. Therefore, these findings should be considered exploratory and hypothesis-generating.

### Integrated perspective on the gut–kidney–immune axis

**Figure 6:**
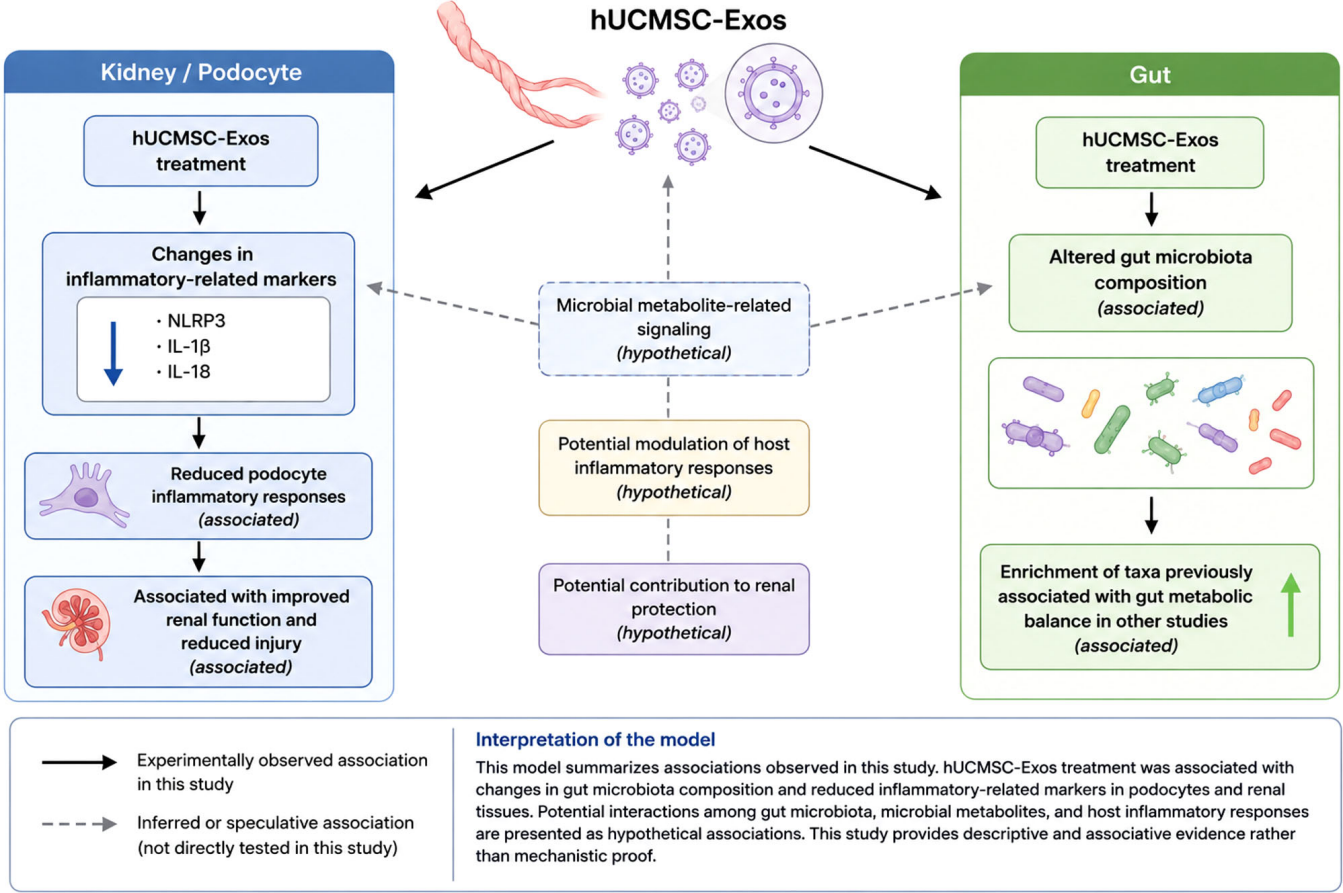
Proposed association model of hUCMSC-Exos in IgAN-like mice. Schematic illustration summarizing the associations observed in this study. hUCMSC-Exos treatment was associated with altered gut microbiota composition and reduced inflammatory-related markers in podocytes and renal tissues. Potential interactions among gut microbiota, microbial metabolites, and host inflammatory responses are presented as hypothetical associations. Solid arrows indicate experimentally observed associations, whereas dashed arrows indicate inferred or speculative relationships that were not directly tested in this study.

Taken together, our findings support a model in which hUCMSC-Exos may influence both local renal inflammation and systemic microbial ecology (**Figure 6**). Rather than acting through a single linear pathway, these effects are more likely to reflect parallel and potentially interacting processes, including modulation of podocyte inflammatory responses and alterations in gut microbiota composition. This integrated perspective aligns with the emerging concept of a gut–kidney–immune axis in chronic kidney diseases (6, 7, 13). However, the causal relationships among these components remain to be fully elucidated. Recent integrated multi−omics approaches have successfully unveiled microbiota−metabolite−host interactions and identified novel biomarkers for early diabetic kidney disease (44). Our study is limited to 16S rRNA sequencing and bulk transcriptomics, which cannot capture functional metabolites or cell−type−specific responses. Future studies should combine metagenomics, metabolomics (e.g., SCFAs, tryptophan metabolites), and single−cell transcriptomics to establish causal links in IgAN.

### Limitations

Several limitations of this study should be acknowledged. First, causal relationships between gut microbiota alterations and renal outcomes were not established. Second, microbial metabolites such as SCFAs were not directly measured, and multi-omics approaches, including metagenomics and metabolomics, were not performed, limiting functional interpretation of microbiome changes. Third, mechanistic validation of the AhR signaling pathway was not performed, and its involvement was inferred solely from transcriptomic data. Fourth, key markers of NLRP3 inflammasome-related responses (cleaved caspase-1, ASC oligomerization, GSDMD cleavage) were not assessed. Fifth, podocyte experiments did not include healthy human IgA1 controls; therefore, the specificity of Gd-IgA1–induced responses could not be fully distinguished from nonspecific IgA-mediated effects. Sixth, the active cargo of hUCMSC-Exos (e.g., miRNAs or proteins) was not identified. Seventh, detailed characterization of exosomes (e.g., endotoxin levels and particle-to-protein ratio) was not conducted, which may influence interpretation of biological activity. Eighth, the IgAN mouse model does not fully recapitulate the mucosal immune origin of human disease, and histopathological assessment was based on representative images without quantitative scoring, which may limit robustness and reproducibility. Ninth, GSEA was not performed on the GSE93798 dataset due to incompatibility of the available data format with standard GSEA pipelines. This study provides descriptive and associative evidence rather than mechanistic proof, and should be interpreted in this context.

## Conclusion

These findings are consistent with the emerging concept of a gut–kidney–immune axis in IgAN and generate hypotheses for further investigation of extracellular vesicle-based interventions. However, causal relationships between gut microbiota alterations, inflammasome-related signaling, and renal protection cannot be established from the current data. Functional validation of microbial metabolites, inflammasome activation pathways, and exosome cargo was not performed. Therefore, the proposed biological interpretations should be considered hypothesis-generating rather than mechanistic conclusions. Future studies incorporating causal microbiome interventions, targeted metabolomic profiling, and pathway-specific functional experiments will be required to clarify the role of hUCMSC-Exos in IgAN pathophysiology.

## Data Availability

Raw 16S rRNA sequencing data have been deposited in the NCBI Sequence Read Archive (SRA) under BioProject accession PRJNA1462302 (SRA submission: SUB16165529). The data will be publicly available upon publication. The GSE93798 dataset, comprising glomerular transcriptomic profiles from IgAN patients and controls, is publicly available from the Gene Expression Omnibus under accession number GSE93798. Uncropped Western blot images are provided in Supplementary File 1. Other data are available from the corresponding author upon reasonable request.

## Supplementary material

Supplementary Figure 1 Characterization of hUCMSCs and hUCMSC-Exos. **(A)** Morphology of hUCMSCs under light microscopy (scale bar: 50 μm). **(B)** Flow cytometric analysis showing expression of mesenchymal markers (CD73, CD90, CD105) and absence of hematopoietic marker CD45. Numbers indicate the percentage of positive cells relative to isotype controls. **(C)** Transmission electron microscopy (TEM) image of hUCMSC-Exos (scale bar: 100 nm). **(D)** Nanoparticle tracking analysis (NTA) showing size distribution of hUCMSC-Exos (mean diameter: 75.5 nm; concentration: 7.63×10^9^ particles/mL). **(E)** Western blot analysis showing ALIX, Calnexin, CD81, and CD9 are exosomal markers, while Calnexin is a negative marker. Molecular weight markers (kDa) are indicated. Uncropped full−membrane images are provided in Supplementary File 1.

Supplementary Figure 2 Rarefaction curves of observed ASVs. Rarefaction curves based on observed ASVs at the ASV level. The curves plateaued after approximately 20,000 reads per sample, indicating sufficient sequencing depth. Each line represents the mean of all samples within the group (n=5 per group).

Supplementary Figure 3 Alpha diversity indices. **(A)** Shannon index; **(B)** Chao1 index. No significant differences were observed among the three groups (Kruskal-Wallis test, Shannon: P = 0.2982; Chao1: P = 0.4025). Boxes represent the interquartile range, horizontal bars indicate the median, and whiskers show the minimum and maximum values.

Supplementary Table 1 Differentially abundant bacterial genera among groups (Kruskal-Wallis test with FDR correction). Genera with nominal P < 0.10 are shown to illustrate trends; no genus reached significance after FDR correction (all q > 0.05). Relative abundances (%) are presented as mean ± SD (n=5 per group). P-values were obtained from Kruskal-Wallis tests. FDR q-values were calculated using the Benjamini-Hochberg method. The lack of significance after correction is likely due to the limited sample size and high within-group variability.

Supplementary Table 2 Pairwise PERMANOVA of gut microbiota composition among groups. Pairwise PERMANOVA based on Bray–Curtis distance was performed with 9,999 permutations. Bonferroni-adjusted P values are shown (significance threshold α = 0.05/3 = 0.0167). Significant difference was observed between Control and IgAN (adjusted P = 0.03). The comparison between IgAN and IgAN+hUCMSC-Exos showed a non-significant trend (adjusted P = 0.066). No significant difference was observed between Control and IgAN+hUCMSC-Exos (adjusted P = 1.0). Analyses were performed using the Shanghai Majorbio cloud platform.
