# Supplementary figures and images for: Human umbilical cord mesenchymal stem cell–derived exosomes are associated with changes in renal injury markers, gut microbiota composition, and inflammatory signaling in IgA nephropathy
Source: Front Immunol. 2026 Jun 1;17:1854005. doi: 10.3389/fimmu.2026.1854005 (PMC13265289; doi:10.3389/fimmu.2026.1854005)

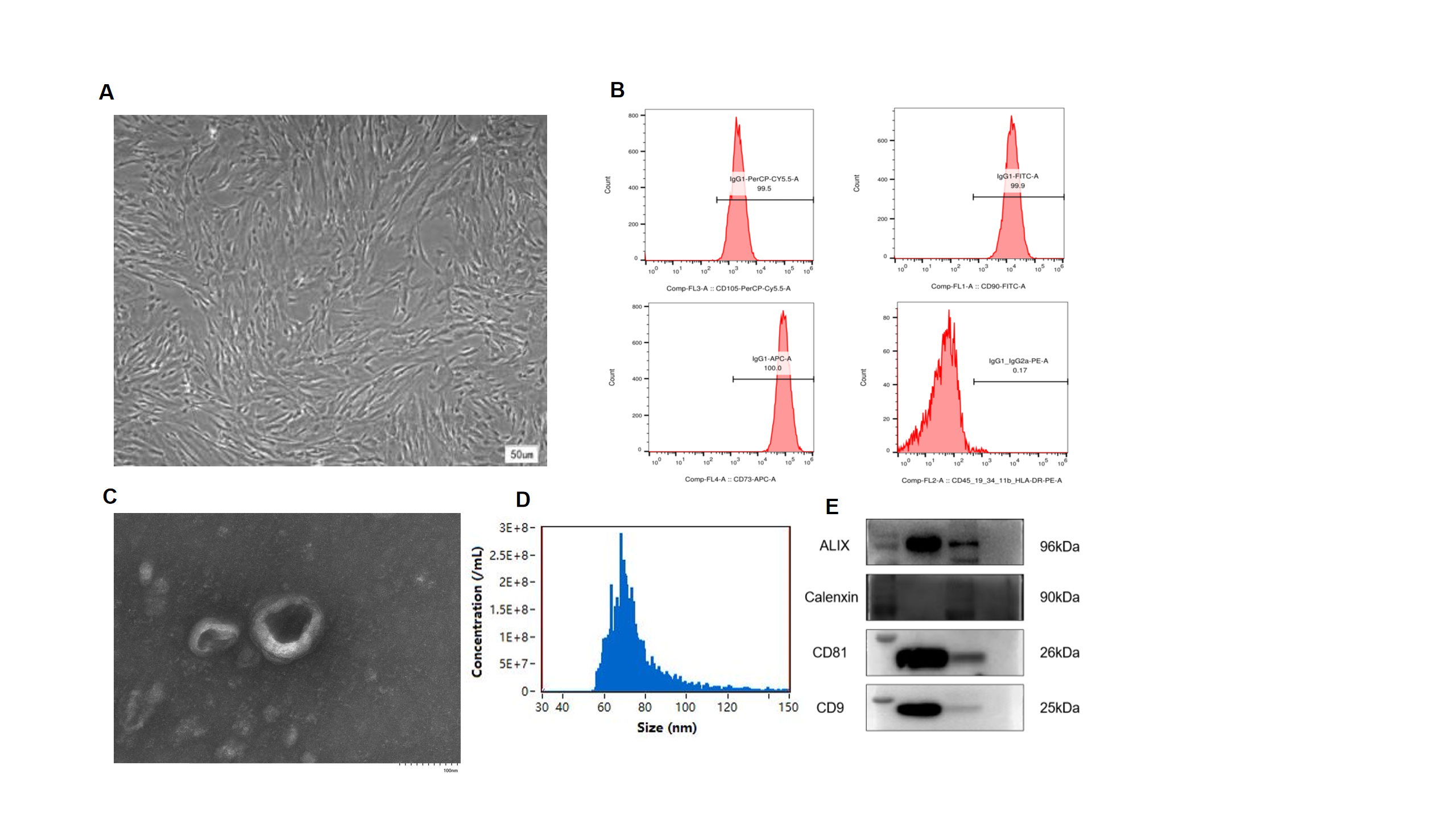

Supplement: Supplementary Figure 1 — Characterization of hUCMSCs and hUCMSC-Exos. (A) Morphology of hUCMSCs under light microscopy (scale bar: 50 μm). (B) Flow cytometric analysis showing expression of mesenchymal markers (CD73, CD90, CD105) and absence of hematopoietic marker CD45. Numbers indicate the percentage of positive cells relative to isotype controls. (C) Transmission electron microscopy (TEM) image of hUCMSC-Exos (scale bar: 100 nm). (D) Nanoparticle tracking analysis (NTA) showing size distribution of hUCMSC-Exos (mean diameter: 75.5 nm; concentration: 7.63×10^9^ particles/mL). (E) Western blot analysis showing ALIX, Calnexin, CD81, and CD9 are exosomal markers, while Calnexin is a negative marker. Molecular weight markers (kDa) are indicated. Uncropped full−membrane images are provided in Supplementary File 1. [file Image1.tif]

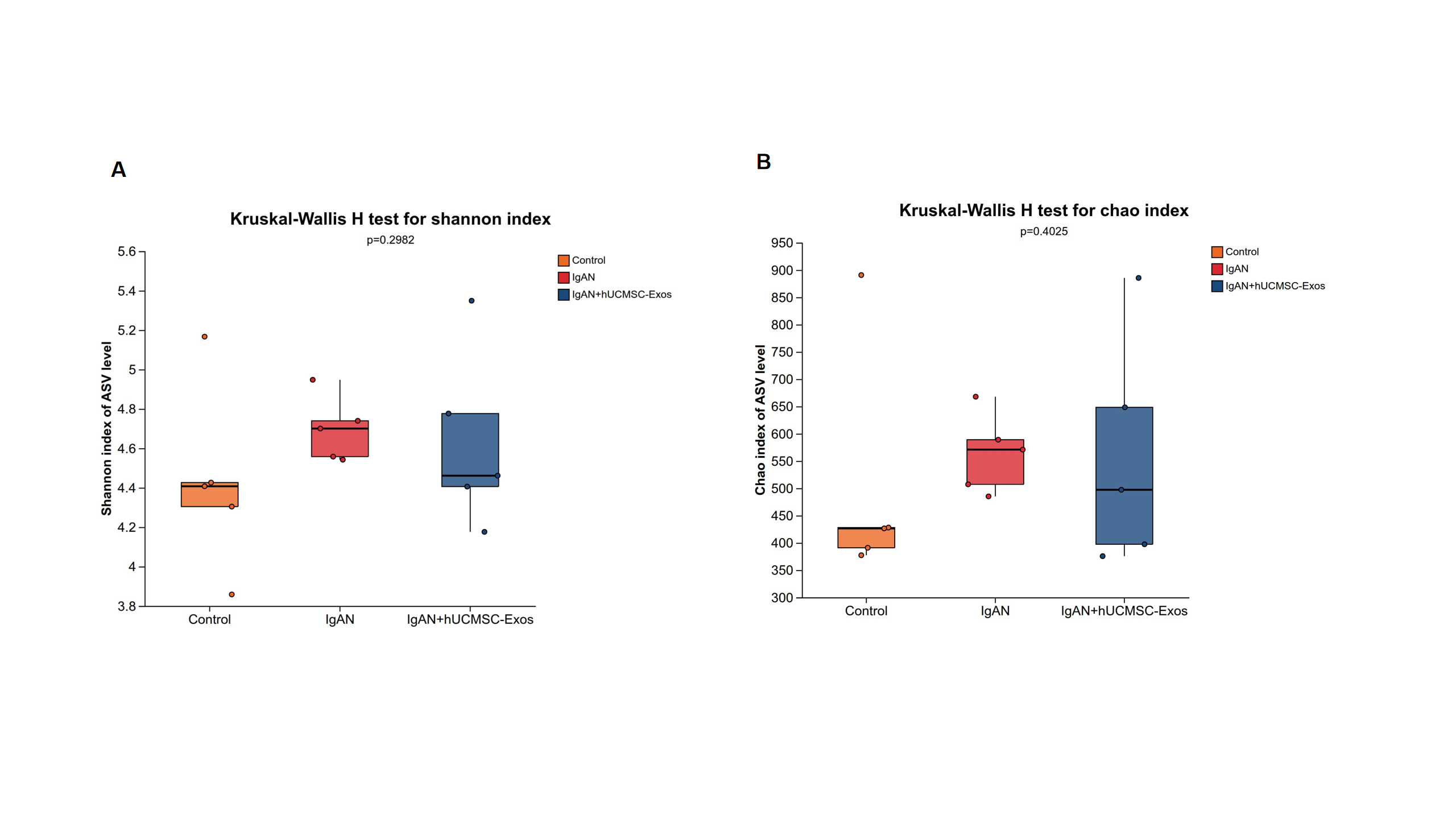

Supplement: Supplementary Figure 3 — Alpha diversity indices. (A) Shannon index; (B) Chao1 index. No significant differences were observed among the three groups (Kruskal-Wallis test, Shannon: P = 0.2982; Chao1: P = 0.4025). Boxes represent the interquartile range, horizontal bars indicate the median, and whiskers show the minimum and maximum values. [file Image3.tif]
