# Supplementary material for: Human umbilical cord mesenchymal stem cell–derived exosomes are associated with changes in renal injury markers, gut microbiota composition, and inflammatory signaling in IgA nephropathy
Source: Front Immunol. 2026 Jun 1;17:1854005. doi: 10.3389/fimmu.2026.1854005 (PMC13265289; doi:10.3389/fimmu.2026.1854005)
Supplement: Supplementary Table 1 — Differentially abundant bacterial genera among groups (Kruskal-Wallis test with FDR correction). Genera with nominal P < 0.10 are shown to illustrate trends; no genus reached significance after FDR correction (all q > 0.05). Relative abundances (%) are presented as mean ± SD (n=5 per group). P-values were obtained from Kruskal-Wallis tests. FDR q-values were calculated using the Benjamini-Hochberg method. The lack of significance after correction is likely due to the limited sample size and high within-group variability. [file Table1.pdf]

Genus	Control (%) mean±SD	IgAN (%) mean±SD	hUCMSC-Exos (%) mean±SD	P-value	FDR q-value
Lactobacillus	17.61 ± 10.01	0.69 ± 0.29	8.77 ± 8.14	0.00701	0.1812
Alloprevotella	2.58 ± 2.88	10.12 ± 5.64	2.79 ± 3.65	0.03652	0.1878
norank_o_Clostridia_UCG-014	2.02 ± 0.51	3.06 ± 1.00	1.69 ± 0.48	0.02990	0.1831
norank_f_Ruminococcaceae	3.41 ± 4.49	0.13 ± 0.07	1.84 ± 1.66	0.01850	0.1831
Parasutterella	1.72 ± 1.13	2.00 ± 0.76	0.46 ± 0.33	0.02065	0.1831
Ruminococcus	0.23 ± 0.44	1.84 ± 1.37	0.36 ± 0.51	0.04372	0.2003
Turicibacter	0.16 ± 0.19	1.91 ± 0.60	0.04 ± 0.02	0.00752	0.1812
Candidatus_Saccharimonas	0.37 ± 0.21	0.20 ± 0.05	1.17 ± 1.18	0.02688	0.1831
Bacillus	1.01 ± 0.58	0.06 ± 0.05	0.48 ± 0.54	0.03496	0.1878
Ileibacterium	0.95 ± 1.11	0.04 ± 0.03	0.12 ± 0.15	0.01120	0.1812
Alistipes	1.11 ± 0.52	2.54 ± 0.86	1.77 ± 1.26	0.07502	0.2701
Corynebacterium	0.01 ± 0.02	1.92 ± 1.93	0.77 ± 1.11	0.05437	0.2175
norank_o_Clostridia_vadinBB60_group	0.10 ± 0.20	0.69 ± 0.06	0.23 ± 0.30	0.01076	0.1812
Enterorhabdus	0.31 ± 0.21	0.06 ± 0.02	0.51 ± 0.40	0.01258	0.1812
Parabacteroides	0.36 ± 0.23	0.33 ± 0.03	0.16 ± 0.08	0.03100	0.1831
norank_f_Mitochondria	0.45 ± 0.54	0.00 ± 0.00	0.27 ± 0.31	0.00726	0.1812
norank_f_UCG-010	0.07 ± 0.07	0.36 ± 0.17	0.25 ± 0.14	0.02589	0.1831
Rikenellaceae_RC9_gut_group	0.07 ± 0.04	0.25 ± 0.07	0.13 ± 0.15	0.04525	0.2003
norank_f_Eubacterium_coprostanoligenes_group	0.12 ± 0.16	0.27 ± 0.21	0.02 ± 0.04	0.00944	0.1812
norank_o_Rhodospirillales	0.01 ± 0.01	0.35 ± 0.28	0.01 ± 0.02	0.00726	0.1812
Clostridium_sensu_stricto_1	0.03 ± 0.06	0.24 ± 0.14	0.06 ± 0.12	0.02668	0.1831
Prevotellaceae_UCG-001	0.01 ± 0.01	0.13 ± 0.06	0.05 ± 0.08	0.04719	0.2003
Paraprevotella	0.01 ± 0.01	0.13 ± 0.07	0.02 ± 0.02	0.00991	0.1812
Parvibacter	0.07 ± 0.05	0.01 ± 0.01	0.08 ± 0.06	0.01753	0.1831
Coriobacteriaceae_UCG-002	0.00 ± 0.00	0.09 ± 0.03	0.06 ± 0.06	0.00592	0.1812
Streptococcus	0.09 ± 0.10	0.02 ± 0.02	0.02 ± 0.02	0.04729	0.2003
Christensenellaceae_R-7_group	0.02 ± 0.02	0.05 ± 0.03	0.01 ± 0.00	0.04107	0.1972
Eubacterium_nodatu	0.01 ± 0.01	0.05 ± 0.03	0.01 ± 0.02	0.01484	0.1831

m_group

Supplementary Table S1. Differentially abundant bacterial genera among groups (Kruskal-Wallis test with FDR correction).

Genera with nominal $P < 0.10$ are shown to illustrate trends; no genus reached significance after FDR correction (all $q > 0.05$). Relative abundances (%) are presented as mean ± SD (n=5 per group). P-values were obtained from Kruskal-Wallis tests. FDR q-values were calculated using the Benjamini-Hochberg method. The lack of significance after correction is likely due to the limited sample size and high within-group variability.
