# Supplementary material for: Human umbilical cord mesenchymal stem cell–derived exosomes are associated with changes in renal injury markers, gut microbiota composition, and inflammatory signaling in IgA nephropathy
Source: Front Immunol. 2026 Jun 1;17:1854005. doi: 10.3389/fimmu.2026.1854005 (PMC13265289; doi:10.3389/fimmu.2026.1854005)
Supplement: Supplementary Table 2 — Pairwise PERMANOVA of gut microbiota composition among groups. Pairwise PERMANOVA based on Bray–Curtis distance was performed with 9,999 permutations. Bonferroni-adjusted P values are shown (significance threshold α = 0.05/3 = 0.0167). Significant difference was observed between Control and IgAN (adjusted P = 0.03). The comparison between IgAN and IgAN+hUCMSC-Exos showed a non-significant trend (adjusted P = 0.066). No significant difference was observed between Control and IgAN+hUCMSC-Exos (adjusted P = 1.0). Analyses were performed using the Shanghai Majorbio cloud platform. [file Table2.pdf]

Comparison	SumsOfSqs	MeanSqs	F.Model	R^2	P-value
Control VS IgAN	0.25743	0.25743	3.34962	0.29513	0.01
Control VS IgAN+hUCMSC-Exos	0.0671	0.0671	0.62361	0.07231	0.599
IgAN VS IgAN+hUCMSC-Exos	0.13411	0.13411	2.88957	0.26535	0.022

Supplementary Table S2. Pairwise PERMANOVA of gut microbiota composition among groups.

Pairwise PERMANOVA based on Bray–Curtis distance was performed with 9,999 permutations. Bonferroni-adjusted P values are shown (significance threshold $\alpha = 0.05/3 = 0.0167$). Significant difference was observed between Control and IgAN (adjusted P = 0.03). The comparison between IgAN and IgAN+hUCMSC-Exos showed a non-significant trend (adjusted P = 0.066). No significant difference was observed between Control and IgAN+hUCMSC-Exos (adjusted P = 1.0). Analyses were performed using the Shanghai Majorbio cloud platform.
